# An Ultrastructural Study on the Effect of High Temperatures on Teeth and Restorative Materials That Aids in the Identification of Human Remains

**DOI:** 10.1155/2021/6629560

**Published:** 2021-07-01

**Authors:** V. Yashoda, Manay Srinivas Munisekhar, S. Shylaja, Krishna A. Rao, Sharath Kumar Reddy, Farahnaz Muddebihal, Mohammad Khursheed Alam

**Affiliations:** ^1^Yashoda Dental Clinic, India; ^2^Oral Pathology and Oral Biology Division, Department of Preventive Dentistry, College of Dentistry, Jouf University, Sakaka, Saudi Arabia; ^3^Department of Oral and Maxillofacial Pathology, SVS Institute of Dental Sciences, Mahabubnagar, India; ^4^Department of Preventive Dentistry, College of Dentistry, Jouf University, Sakaka, Saudi Arabia

## Abstract

**Introduction:**

In most disasters, teeth are the only means of positive identification of an otherwise unrecognizable body, as there has been tremendous increase in the use of dental restorations that have different resistance to prolonged high temperature which is an important aid in identifying burned victims. Application of SEM/EDS in forensics was found useful in areas where there is a need for good imaging with high magnification combined with elemental analysis. The objective of this study was to analyze incineration effects on teeth and restorative materials using SEM/EDS. *Materials and Method*. 128 extracted teeth were collected, 96 were restored with silver amalgam, composite, and GIC of 32 each, and crown preparation was done in 32 teeth for which metal ceramic crowns were prepared. These teeth were subjected to 4 different temperatures (500°C, 700°C, 900°C, and 1100°C) for 20 minutes, and they were analyzed macroscopically and by using SEM for the changes subsequent to their exposure to such high temperatures.

**Results:**

All the restorations which were very difficult to identify by naked eye were identified with the help of SEM/EDS.

**Conclusion:**

Elemental analysis of the specific restorative material proves to be an essential tool for the forensic odontologist.

## 1. Introduction

Personal identification is very much necessary for unknown deceased person in homicide, suicide, accident, mass disasters, etc. Personal identification is also necessary for living individuals who are missing due to amnesia and culprits hiding their identity [1]. Teeth are the most indestructible components of human body. These structures have the highest resistance to most environmental attacks [2]. In mass disasters, it is not only extremely difficult to identify, but it requires skill and profound knowledge of the more apt samples to be collected that may help in identifying the victims from the available remains. Based on the dental remains, in 1897, Dr. Oscar Ameodo was able to identify the victims in a fire accident [3]. However, the margin of error is said to be high by simple visual (naked eye) method [4].

In some situations, offenders may employ individual or common graves, obliterate finger prints, destroy teeth, and cremate the remains to avoid identification of a corpse or human remains. The most usual matching process is conducted based upon dental treatment such as prosthetic restorations and dental fillings. Hence, having a thorough knowledge about the possible changes in the dental materials used to restore dental defects in such victims may serve as a vital aid in identifying burnt victims [2]. Many dental restorative materials like silver amalgam, glass ionomer cement (GIC), and composites are different chemically with distinct physical properties with varied abilities to withstand high temperatures. Due to such fundamental differences, these materials behave and respond differently when subjected to varying levels of heat. Such differences may be used as an aid in identification of exact type of restoration. Due to contrasting and variability in the results of the various studies in the past regarding the changes in the structure of teeth and restorative materials with respect to the variable temperatures, this study was undertaken to note changes along with elemental analysis [5].

Forensic applications of scanning electron microscope (SEM) are found mostly in areas where there is a need for good imaging at relatively high magnifications in combination with elemental analysis. Striking property of SEM is its ability to combine imaging with elemental analysis together. SEM along with EDS (energy dispersive spectrometer) helps in detection of elements [2]. Hence, in this study, SEM is used to identify the incinerated tooth with restorative materials through their chemical composition.

## 2. Materials and Methods

128 extracted teeth were used, and all the teeth collected were extracted for the therapeutic procedures. The teeth used in the study were healthy premolars and molars. The exclusion criteria consisted of the unhealthy teeth. The teeth were grouped into 4, each consisting of 32 teeth based on restorative materials used to restore them, namely, GIC, silver amalgam, composites, and ceramics.

Class I cavities were prepared for 3 groups (96 teeth) with high-speed hand piece which were restored with GIC, silver amalgam, and composite material, respectively. Zinc phosphate base was used for 32 teeth that were restored with silver amalgam. For remaining 32 teeth, crown preparation was done which were restored with metal-ceramic crowns prepared in the porcelain laboratory of our institution.

Further, 32 teeth in each group were subdivided into 4 groups (8 teeth in each group) depending on the temperatures 500°C, 700°C, 900°C, and 1100°C to which they were exposed. Stainless steel capsules were used to place the teeth. Single tooth in each group was incinerated in a burnout furnace. The time taken to reach different temperatures from room temperature was different. For 500°C, it was 1 hour 30 minutes, for 700°C, it was 2 hours, for 900°C, it was 2 hours 30 minutes, and for 1100°C, it was 3 hours. After reaching the set temperatures, they were incinerated for a constant period of 20 minutes.

Subsequently, all the teeth were analyzed for changes macroscopically and by scanning electron microscopy. For SEM analysis, further processing was not done since the teeth were in a desiccated state. Hitachi T-300 SEM at an accelerating voltage of 20 kV was used. SEM for ultrastructural changes of teeth and EDS for chemical analysis of the restorative materials were done.

## 3. Results

### 3.1. Macroscopic Findings

**Figure 1:**
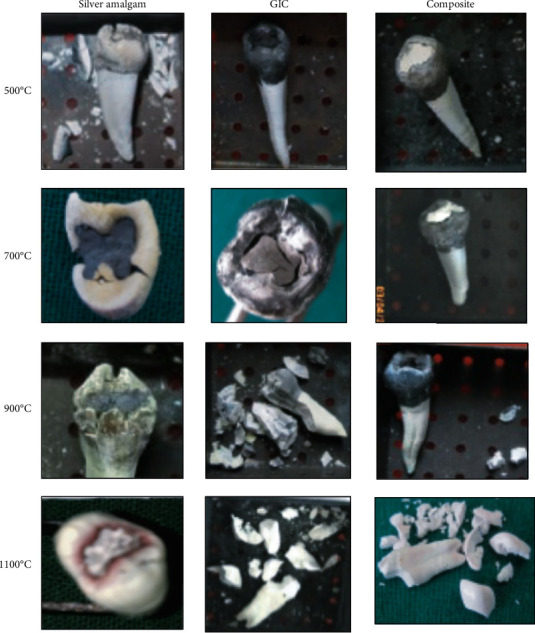
Morphological alterations of restorations in the tooth after incineration.

The changes observed were due to their physicochemical attributes. Postincineration, the most common changes noted macroscopically were a gradual change in the color and morphological structure and appearance of both the teeth and restorative materials (Table 1). The change in the color and external appearance are the most commonly observed changes seen in incinerated restorative materials and teeth. Morphologically, cracks were seen on surface of the teeth at all temperatures. At 500°C, 700°C, and 900°C, some teeth had intact enamel with dentin, whereas at 1100°C, fragmentation of all the teeth has been observed to a great extent (Figure 1).

### 3.2. Ultrastructural Findings of the Structure of Incinerated Tooth at Various Temperatures

#### 3.2.1. At 500°C

In our study at 500°C, prominent fissures were evident on the surface of enamel and deep fissures in the enamel subsurface. Dentin showed clear dentinal tubules with distinct borders and branching. Cementum surface showed numerous rounded projections and prominent fissures.

#### 3.2.2. At 700°C

Even at 700°C, enamel rods and tubular pattern of dentinal tubules were still identifiable in the enamel and dentin, respectively. The cementum surface appeared melted showing granular tissue underneath.

#### 3.2.3. At 900°C

The morphological appearance of enamel rods could be still identified in the enamel, whereas the dentin appeared to be melted with identifiable dentinal tubules. Cementum showed surface cracks and melted appearance.

#### 3.2.4. At 1100°C

The arrangement of enamel rods was visible with the impression of Tome's process, and the surface was granular, while in dentin, the dentinal tubules were obliterated, and the surface of cementum showed granularity and appeared melted.

### 3.3. Elemental Analysis

In our study, when amalgam was analyzed for elements present prior to incineration, the chief components such as silver, tin, mercury, and copper with traces of magnesium and aluminum were detected in all samples. However, when the samples were subjected to various temperatures, the elements that were detected were very similar containing silver, tin, and copper along with zinc and phosphorus.

Since the chief components of amalgam restoration like silver, tin, and copper were present at all temperatures to which the teeth were subjected to, the restoration may be identified as amalgam restoration positively. However, at the temperatures beginning from 500°C, mercury was not detected as it assumed to have evaporated as vapors (Table 2).

For GIC restoration, elemental analysis of preincinerated tooth showed the presence of silicon, aluminum, calcium, molybdenum, and sodium. It was observed that after incineration at different temperatures, the restorations showed the presence of major elements like silicon, aluminum, calcium, and sodium, and the restorations were identified as GIC accurately (Table 2).

Elemental analysis of composite restorations prior to incineration detected various elements like silicon, aluminum, iron, and barium. After exposure of teeth with composite to high temperatures, the elements detected were variable at different temperatures. At 500°C, Si, Ba, Al, Ca, and P were detected, whereas at 700°C, presence of Si, Al, Ti, Ca, P, and Ni was detected. At 900°C and 1100°C, along with Si, Ba, Al, and Ca, additional elements like tungsten and nickel were identified, respectively (Table 2).

Elemental analyses of the metal the metal portion of all metal ceramic crowns showed Cr, Al, and Ni, while the ceramic portion showed elements such as Si and Al, respectively, before incineration. Though the elemental analysis of metal portion of the crown at 500°C, 900°C, and 1100°C revealed Cr, Al, Ni, and Si, at 700°C, it showed only Cr, Ni, and Si and elemental analysis of the ceramic portion at 500°C Si, Na, S, and Br, at 700°C, 900°C, and at 1100°C Si, Al, Na, and K, respectively (Table 3).

## 4. Discussion

Forensic odontology is an application of dental science in legal investigations, in both identification of the offender and the victim based on dental records. In addition, in case of mass disaster identification of the teeth and dental records seems to be the one among the most reliable approaches for human identification. One of the methods for identification is to collect samples from the human remains including the teeth with or without restorations and analyze the changes in teeth and dental materials subjected to high temperature [6].

The temperatures to which the victims are subjected to in fire accidents depend upon a variety of factors such as the place (open or closed environment), duration of time period to which they are exposed to fire, type of oxidant, and various extinguishing agents which might also influence their effects on teeth and restorations.

While handling incinerated teeth, adequate caution must be observed to strengthen the burnt remains and to prevent further disintegration and decomposition. One of the most common issues is fragmentation of burnt remains. Gustafson et al. have reported that no significant changes are noticed at temperatures below 200°C. Bodies might be exposed to different range of temperatures during such mishaps. While the temperatures may be 1200°F (649°C) in case of house fires, it may be between 871 and 982°C during cremation and between 1600 and 1800°F (800 and 1100°C) when petrol is used. Therefore, in our study, the teeth were subjected to temperatures ranging between 500 and 1100°C. The separation of restorations from the cavity walls with resultant microleakage is attributed to the loss of mercury and organic matrix [7].

When subjected to high temperatures suddenly, it results in rapid carbonization of the teeth which may be related to the charred appearance of the teeth. In the present study, the teeth were restored after extraction, and hence, time elapsed between the filling of the tooth and fire accident could not be included to analyze if there were any differences in the macroscopic changes and elemental analysis depending upon the varied durations of the fillings done. Some teeth with fillings in all the four groups were unscathed even at 900°C which was in accordance with the study done by Karkhanis et al. [7]. Such a finding could be attributed to incorporation of retention form while preparing the cavity. Cracks and fissures were noted in teeth with restorations at all temperatures. This might possibly be due to a disturbance in the structural integrity of the tooth, while cavities were being prepared. Ceramic crowns could be identified visually as the crowns were prepared at these high temperatures. However, they got separated from the teeth.

The results of the present study were consistent with the study of Moreno et al. who have observed similar pink pigmentation at 1000°C in both crown and roots. The pink pigmentation of the tooth at high temperature was attributed to oxidation of copper present in the amalgam restoration to copper oxide. The possibility of such phenomenon may be true as the present elemental analysis has shown the presence of copper at 1100°C [8]. However, the results of the present study were not in accordance to few other studies by Gunther and Schmidt and Vazquez et al. who have reported quite different results as compared to the present study. Formation of golden thread surrounding the restoration from 800°C to 1200°C was observed by them. It was explained that such a change was due to mercuric oxide vapors in reddish and yellowish tones [9]. We differ from such an explanation as in the present study, in all, the samples from 500°C onwards and elemental analysis did not show any presence of mercury. Thus, the absence of golden thread formation at all temperatures in this study is justified.

The changes noted in the ceramic crowns exposed to high temperatures were minimal as they are prepared at very high temperatures like 900°C. All the crowns got dislodged from the teeth which could be attributed to the shrinkage of tooth root and crown due to loss of organic matrix. At 1100°C, the architecture on the occlusal surface of ceramic crown was lost which was consistent with a study by *Patidar* et al. who opined that it could be due to ceramic flow on the surface [10]. However, in another study by Khirtika and Ramesh, the results were slightly different as the temperatures at which the teeth along with crowns were different, and secondly, in their study, endodontically treated teeth with all-ceramic crowns were used [11].

Fragments of incinerated teeth under SEM revealed remnants of restorative materials and indentations of bur used. These pictures also showed the presence of dentinal structures indicating the high resistance of these structures to extreme heats. Uniform changes were not seen ultrastructurally in enamel and dentin which was similar to observations of Wilson and Massey [12], whereas Karkhanis and Franklin had seen star-shaped crystals originating from peritubular dentin which was not found in this study. So, we cannot rely only on SEM for positive identification of victims [7].

Many studies have been done for analyzing the changes in incinerated teeth and restorative materials where it was compared only by visual examination. Visual examination includes comparison of color changes and presence of cracks and fissures. Such findings were also not consistent with the rise of temperature and hence may not be helpful for positive identification of the restorations. Hence, in our study EDS system was employed for analyzing various elements in the restorations to identify the restorative material more accurately.

To the best of our knowledge, till now, there are no studies where the elemental analysis of amalgam, composite, and GIC restorations was done after incinerating at high temperatures to compare. However, Bonavilla and Moreno Gomez in their studies used SEM/EDS to analyze the effects of high temperatures on root canal filling materials where they positively identified the materials through elemental analysis strengthening the present view that elemental analysis of the restorative materials could serve as an effective tool in identification in forensics [13, 14]. In another study, though Pol and Gosavi have studied the effect of temperatures ranging from 200°C to 1000°C (200°C, 400°C, 600°C, 800°C, and 1000°C) on teeth and restorations including all ceramic crowns on their structure using SEM, elemental analysis was done [15].

All the restorations in our study were identified with the help of SEM/EDS. Elemental analysis of the specific restorative material proves to be an essential tool for the forensic odontologist.

## 5. Conclusion

SEM/EDS might be employed to identify an individual positively by analyzing the various elements present in the dental remains which have been incinerated to the point where their fillings are highly fragmented by the intense heat. Though SEM has been helpful in identifying individuals from teeth, in those teeth with restorations, SEM examination along with elemental analysis may further enhance the positive identification.

## Figures and Tables

**Table 1:** Color changes in teeth and restorations after incineration.

	500°C	700°C	900°C	1100°C
Enamel	Brown to grey	Grayish black	Grayish white	Chalky white
Dentin	Grey	Grey	Ash to white	White
Cementum	White	White	White	White
Silver amalgam	Grey	Grey	Grey	Grey
Zinc phosphate	White with brown margins	White	White	Grey
GIC	Grayish black	Grey	Grey	White
Composite	White	White	White	White

**Table 2:** Showing elemental analysis of restorations before and after incineration.

	GIC	Composite	Silver amalgam
Preincineration	Si, Al, Ca, Mo, Na	Si, Al, Fe, Ba	Sn, Ag, Hg, Cu, Mg, Al
500°C	Al, Si, Sr, Ca, P, Na	Si, Ba, Al, Ca, P	Sn, Ag, Cu, Zn, P
700°C	Ca, Al, Sr, Si, P, Na,	Si, Al, Ti, Ca, P, Ni	Sn, Ag, Cu, Zn, P
900°C	Al, Sr, Si, Ca, P, Na	Si, Ba, Al, Ca, Ni, W	Sn, Ag, Cu, Zn, P
1100°C	Sr, Al, Si, P, Ca, Na	Si, Al, Ba, Ca, Ni, P	Sn, Ag, Cu, Zn, P

**Table 3:** Elemental analyses of ceramic crowns before and after incineration.

	Metal part	Ceramic part
Preincineration	Cr, Al, Ni	Si, Al
500°C	Cr, Al, Ni, Si	Si, Na, S, Br
700°C	Cr, Ni, Si	Si, Al, Na, K
900°C	Cr, Ni, Al, Si	Si, Al, Na, K
1100°C	Cr, Ni, Al, Si	Si, Al, Na, K

## Data Availability

Details are presented within the article in the form of tables and text in results. Other data will be made available upon request.

## References

[1] Ubelaker D. H. (2009). The forensic evaluation of burned skeletal remains: a synthesis. *Forensic Science International*.

[2] Valenzuela A., Martin-de las Heras S., Marques T., Exposito N., Bohoyo J. M. (2000). The application of dental methods of identification to human burn victims in a mass disaster. *International journal of legal medicine*.

[3] Jayakrishna Babu P., Krishna Mohan T., Jyothi A. (2012). Role of dentists in manmade disasters: a review. *Indian J of Forensic Odontology*.

[4] Sweet D. (2010). Forensic dental identification. *Forensic Science International*.

[5] Whittaker D. K., MacDonald D. G. (1989). *A Color Atlas of Forensic Dentistry*.

[6] Ermenca B., Renerb K. (1999). Possibilities for dental identification in the case of mass disaster in Slovenia. *Forensic Science International*.

[7] Karkhanis S., Ball J., Franklin D. (2009). Macroscopic and microscopic changes in incinerated deciduous teeth. *Journal of Forensic Odonto-Stomatology*.

[8] Moreno S., Merlati G., Marín L., Savio C., Moreno F. (2009). Effects of high temperatures on different dental restorative systems: experimental study to aid identification processes. *Journal of Forensic Dental Sciences*.

[9] Vázquez L., Rodríguez P., Moreno F. (2012). *In vitro* macroscopic analysis of dental tissues and some dental materials used in endodontics, submitted to high temperatures for forensic applications. *Revista Odontológica Mexicana*.

[10] Patidar K. A., Parwani R., Wanjari S. (2010). Effects of high temperature on different restorations in forensic identification: dental samples and mandible. *Journal of Forensic Dental Sciences*.

[11] Khirtika S. G., Ramesh S. (2017). Effect of high temperature on crowns as post-endodontic restoration in forensic analysis: an in vitro study. *Journal of Advanced Pharmacy Education & Research*.

[12] Wilson D. F., Massey W. (1987). Scanning electron microscopy of incinerated teeth. *The American Journal of Forensic Medicine and Pathology*.

[13] Bonavilla J. D., Bush M. A., Bush P. J., Pantera E. A. (2008). Identification of incinerated root canal filling materials after exposure to high heat Incineration. *Journal of forensic sciences*.

[14] Freddy M. G., Carlos M. P. (2011). Scanning electron microscopy analysis of two endodontically treated teeth subjected to high temperatures. A pilot study. *Revista Facultad de Odontología Universidad de Antioquia*.

[15] Pol C. A., Gosavi S. R. (2014). Scanning electron microscopic analysis of incinerated teeth: an aid to forensic identification. *Journal of oral and maxillofacial pathology*.

